# A new two-stage method for revealing missing parts of edges in protein-protein interaction networks

**DOI:** 10.1371/journal.pone.0177029

**Published:** 2017-05-11

**Authors:** Wei Zhang, Jia Xu, Yuanyuan Li, Xiufen Zou

**Affiliations:** 1 School of Science, East China Jiaotong University, Nanchang 330013, China; 2 School of Mechatronic Engineering, East China Jiaotong University, Nanchang 330013, China; 3 School of Mathematics and Statistics, Wuhan Institute of Technology in Wuhan, Wuhan, 430072, China; 4 School of Mathematics and Statistics, Wuhan University, Wuhan 430072, China; Weizmann Institute of Science, ISRAEL

## Abstract

With the increasing availability of high-throughput data, various computational methods have recently been developed for understanding the cell through protein-protein interaction (PPI) networks at a systems level. However, due to the incompleteness of the original PPI networks those efforts have been significantly hindered. In this paper, we propose a two stage method to predict underlying links between two originally unlinked protein pairs. First, we measure gene expression and gene functional similarly between unlinked protein pairs on Saccharomyces cerevisiae benchmark network and obtain new constructed networks. Then, we select the significant part of the new predicted links by analyzing the difference between essential proteins that have been identified based on the new constructed networks and the original network. Furthermore, we validate the performance of the new method by using the reliable and comprehensive PPI dataset obtained from the STRING database and compare the new proposed method with four other random walk-based methods. Comparing the results indicates that the new proposed strategy performs well in predicting underlying links. This study provides a general paradigm for predicting new interactions between protein pairs and offers new insights into identifying essential proteins.

## Introduction

With the rapid development of modern high-throughput technologies such as yeast two-hybrid (Y2H)screens [1, 2], tandem affinity purification (TAP) [3], and mass spectrometric protein complex identification (MS-PCI) [4], large scale PPI data are available for many organisms. PPI networks provide a comprehensive view of the global interaction structure of an organism’s proteome, as well as detailed information on specific interactions, which provide unprecedented opportunities for both biological and computational scientists to understand the cell at a systems level [5]. For example, PPI networks are widely used for predicting protein complexes or functional modules [6, 7], as well as essential proteins [8, 9] and proteins associated with a certain complex disease [10].

However, the growing size and complexity of experimental data obtained from high throughput technologies are incomplete, and the PPI network we obtained through high-throughput technology is far from complete; only a fraction of true PPIs have been documented even for well-known species [11]. The incompleteness of the PPI network will severely impair the prediction precision. Revealing the unknown part of these networks by biological experimental methods is time-consuming and expensive.

In recent years, many computational methods have been proposed to predict the underlying links between two original unlinked proteins [12–14]. These methods basically fall into two categories: topological-based and similarity of protein biological attributes-based methods. The first type of method is based on topological properties such as measuring topological similarity [14–16] and characterizing the ‘distance’ by random walks [17–19]. The second category consists of methods that based on sequence homology as well as protein three-dimensional structural and phylogenetic profiles [20, 21]. Although these methods have improved the accuracy in link prediction, most of these methods highly depend on the topological properties of the original PPI networks and few methods have examined the co-expression and co-functional the between two protein pairs being considered.

In the present study, we present a novel two-stage method for predicting missed links in the PPI networks. First, the Pearson correlation coefficient (PCC) and Gene Ontology (GO) similarity value are used as local similarity indices to predict the existence of links between two unlinked proteins, and we obtained the new constructed networks. Then, we evaluate the new constructed network and the original network by identifying essential proteins and collect the new predicted edges associated with the essential proteins that were neglected in the original network but that are significantly ranked in the top of the new constructed networks. Finally, we assume that the new predicted edges we selected are truly predicted and validate the new predicted edges using the PPI dataset obtained from the database STRING, which is a comprehensive and reliable database. Our findings suggest a hypothesis for predicting interactions between two unlinked protein pairs using a two-stage method.

## Methods

In this section, we first present a new strategy for obtaining a new PPI network by PPI prediction based on gene expression profiles and GO annotation data. Then, we compare the performance of six widely used methods in identifying essential proteins based on new constructed PPI networks and original networks and select the significant part of the new predicted links. Finally, we assume the selected significant parts of the links as the true predicted links and confirm these links by mining the reliable links obtained from the STRING database. Four other random-walk based link prediction methods are used to compare the efficiency of the new strategy.

### Evaluating the existence likelihood between two unlinked proteins

In a PPI network, the weight between two proteins is typically a confidence score of the interaction, represent the probability of the interaction [22]. We assume that the larger similarity weights between proteins indicate a higher probability of the two proteins physically interacting.

Given a PPI network with *N* proteins, we represent the PPI network with an undirected graph *G* = (*V*, *E*), where *V* and *E* are the sets of nodes and links, respectively. To measure the missing links and predict their weight, for each unlinked protein pair (*u*, *v*), we assign a similarity value to quantify the existence likelihood of the link (*u*, *v*). So that all unlinked pairs are ranked according to their value, the top ranked links with larger scores can be considered as the ones with higher existence likelihoods.

*PCC* is a widely used measure the strength of correlation between two variables of linear dependence. To assess the similarity value of the unlinked protein pairs (*u*, *v*), we adapt the *PCC* measurement to evaluate the co-expression value of protein pairs. The *PCC* of a pair of genes (*X* and *Y*) is defined as:

$$\begin{array}{c} PCC\left(X,Y\right)=\frac{1}{n-1}\sum_{i=1}^{n}\left(\frac{X_{i}-mean\left(X\right)}{std(X)}\right)\left(\frac{Y_{i}-mean\left(Y\right)}{std(Y)}\right) \end{array}$$ (1)

where *n* is the number of samples of gene expression data, and *X*_*i*_ is the expression level of gene *i*. The *PCC* of a pair of proteins (*u* and *v*) is defined as the same as the *PCC* of their corresponding gene pairs. The value of PCC ranges from -1 to 1, and the larger *PCC* between the two considered proteins, *u* and *v*, suggests that they are more likely to be co-expressed and interact physically.

Since the physically interacting protein pairs are likely to have the same function, GO annotation provides valuable information for describing biological properties of the gene product and a convenient way to study gene functional similarity. GO has been used as an indicator of the existence likelihoods of the link between two proteins and the GO similarity between interacted protein pairs is higher than disconnected protein pairs [23].

To quantify the functional similarity between two considered proteins, we adapt the GO similarity method proposed in [24] to compute the semantic similarity between GO terms annotated to unlinked protein pairs. The GO similarity between two connected proteins is defined as:

$$\begin{array}{c} GO\_sim\left(u,v\right)=\frac{\sum_{t\in T_{u}\cap T_{v}}\left(S_{u}\left(t\right)+S_{v}\left(t\right)\right)}{\sum_{t\in T_{u}}S_{u}\left(t\right)+\sum_{t\in T_{\text{v}}}S_{v}\left(t\right)} \end{array}$$ (2)

where *S*_*u*_(*t*) is the S-value of GO term *t* related to term *u* and *S*_*v*_(*t*) is the S-value of GO term t related to term *v*.

The GO consists of three sub-ontologies (Biological Process (BP), Cellular Component (CC), and Molecular function (MF)) [25, 26]. The three GO terms are widely used in predicting gene functional associations, and the semantic similarity is used as an indicator for the existence likelihoods of an unlinked edge.

### Another link prediction method

To demonstrate the efficiency of the new proposed strategy, we compare it with four other state-of-the-art link prediction methods on the test PPI network. The first method is the Random Walk with Resistance (RWS) proposed by Lei et al. [14]; the second method is the Local Random Walk (LRW) [27]; the third method is Supervised Random Walk(SRW) developed by Backstorm et al. [18] and the last method is Random Walk with Restart (RWR) [19]. The four methods have been shown to perform well in link prediction for complex networks.

In the present study, we apply the four methods on the test network and obtain the similarity matrix. Then, the similarity values of the unlinked edges are sorted in descending order. To ensure that the number of new predicted links is similar with each other, we set the appropriate threshold value for each of these methods and select the same proportion of these top ranked edges as the new predicted interactions. The steps of random walks in LRW and SRW methods are set to 3, and parameters are set to 0.8.

### Experimental data

To evaluate the performance of the six methods for the new network obtained from link prediction and the original network, we focus our analysis on the widely used Saccharomyces cerevisiae’s PPI data. The first PPI data were downloaded from the DIP database [28]. There is a total of 5093 proteins and 24743 interactions after filtering the duplicate interactions and self-interactions (S1 Text). The second PPI data were obtained from [29], which contains 17201 interactions among 4928 proteins (S2 Text). The third PPI data were described in the published work [7], which contains 14317 interactions and 3672 proteins (S3 Text).

The gold standard essential protein set contains 1285 essential proteins collected from several databases, such as MIPS [30], SGD [31], DEG [32], and SGDP (http://www-sequence.stanford.edu/group/). Out of all the 5093 proteins in the 24743_PPI network, 1167 proteins are essential, 3591 proteins are non-essential, and the remaining 335 proteins have not yet been identified as either essential or non-essential. In the 17201_PPI dataset, 1150 out of 4928 proteins are essential, and the rest are non-essential. In the 14317_PPI dataset, 929 out of 3672 proteins are essential, and the rest proteins are assumed as non-essential.

The gene expression data of Saccharomyces cerevisiae were obtained from the published work in [33], and this dataset contains 36 samples with 6777 genes.

The gene ontology annotations data of Saccharomyces cerevisiae gene products were downloaded from the Gene Ontology Consortium (http://geneontology.org/page/download-annotations). The annotation data for Saccharomyces cerevisiae were released on March 5-th 2016. The GO semantic similarity between two proteins is evaluated by the method mentioned in section 2.1. For proteins that have no corresponding GO id information, we simply set the similarity of the interactions with zero values.

## Results and discussion

We established a general framework to reveal the missed links of the original network by combining the co-expressed measure of gene expression data and GO similarity of GO annotation information. Then, we prune the predicted links and select the most significant links as the true predicted links by filtering the links associated with the essential proteins that could only be predicted under new constructed networks. The selected links are assumed to be true predicted links.

**Fig 1:**
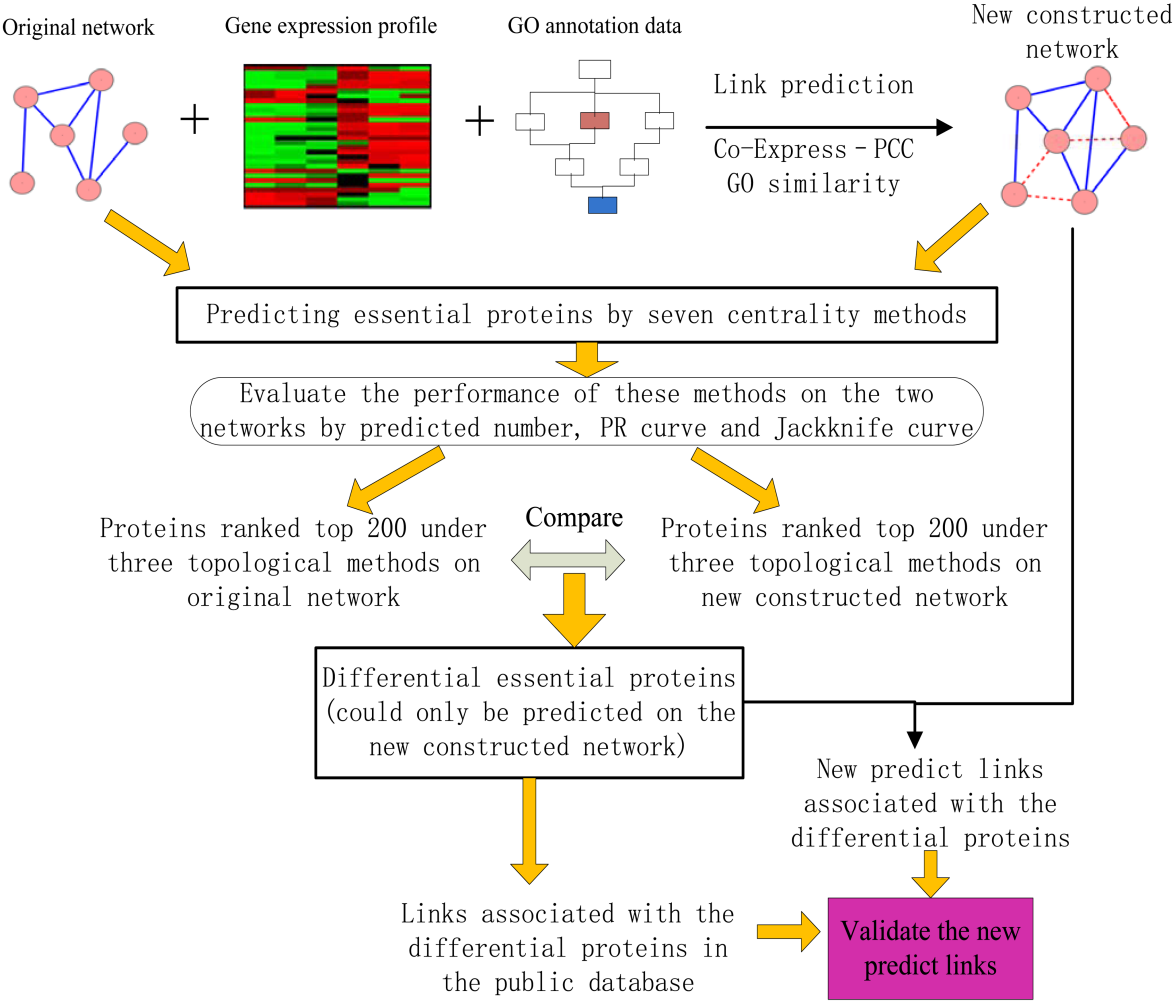
Overview of the proposed strategy for predicting missed links.

Finally, the new predicted links associated with the selected proteins are validated by the reliable links obtained from the public database. The proposed paradigm is depicted in Fig 1.

### Predict the interactions between two unlinked protein pairs and create a new constructed network

In predicting new interactions, we measured the existence likelihood of unlinked protein pairs by measuring the co-expression value and GO similarity value under BP term. The reliability of the new predicted links depends on the threshold of these measures. To obtain appropriate thresholds of PCC and GO similarity, we first collect the number of the links under different thresholds of PCC and then filter the new added links by GO similarity threshold.

Table 1 shows the proportion of the added number of links under the different thresholds of PCC coefficient. When the threshold is set to 0.95, the proportion of the added number of links reached over 80% of the original number of links. The PCC measured the co-expression of gene pairs, which may not guarantee the existence of a physical link between the unlinked protein pairs. To filter the unreliable links, we added GO similarity threshold based on the added links under a different PCC threshold. The GO similarity threshold is set according to the added proportion in Table 1. When the PCC threshold is set relatively low, the GO similarity threshold should increase, so that the quality of predicted links can be guaranteed.

**Table 1:** The proportion of added links under different PCC threshold for 24743_PPI dataset.

PCC threshold	0.95	0.96	0.97	0.98	0.99
Added Proportion	0.815	0.548	0.319	0.133	0.019

Table 2 shows the proportion of new predicted links when both PCC and GO similarity are satisfied. From Table 2, we can see that when the PCC threshold is set to 0.95 and GO similarity threshold is set to 0.5, the proportion of added links is approximately 13.4%, suggesting that adding the GO similarity threshold filtered almost 68% of new added links.

**Table 2:** The proportion of added links under different PCC and GO similarity thresholds for the 24743_PPI dataset.

PCC & GO_sim	0.95 & 0.5	0.96 & 0.4	0.97 & 0.3	0.98 & 0.2	0.99 & 0.1
Added Proportion	0.13449	0.09629	0.06159	0.0289	0.0043

To ensure the reliability of the new predicted links between protein pairs, when the threshold of PCC is set relatively low, the threshold of GO similarity score should be increased, so that the links with a larger similarity score than the threshold can be considered as the ones with high existence likelihood. We use a moderate threshold for PCC as well as for GO similarity when the threshold is too high, few links satisfy the condition and there is no difference between the new constructed network and the original network, and when the threshold is too low, the unreliability o the new constructed network decreases.

In the present study, we illustrated the performance of the strategy by setting the PCC threshold set at 0.98 (refer to network 1 (S4 Text)), or the PCC threshold was set at 0.95, and GO similarity threshold set at 0.5 (refer to network 2 (S5 Text)). The proportion of added links was approximately 13% under both cases for 24743_PPI dataset. In the following analysis, we take the two new constructed networks (network 1 and network 2) as test networks and compared their performance with the original network under each of the considered methods.

### Compare the performance of centrality measures on the original network and new constructed networks

To validate the efficiency of the proposed strategy, we compare it with six centrality methods (the definition of centrality measures is provided in the S1 Appendix) on the new constructed networks and the original network under the benchmark essential protein set. Proteins are sorted in descending order according to their measurements computed under each method.

**Fig 2:**
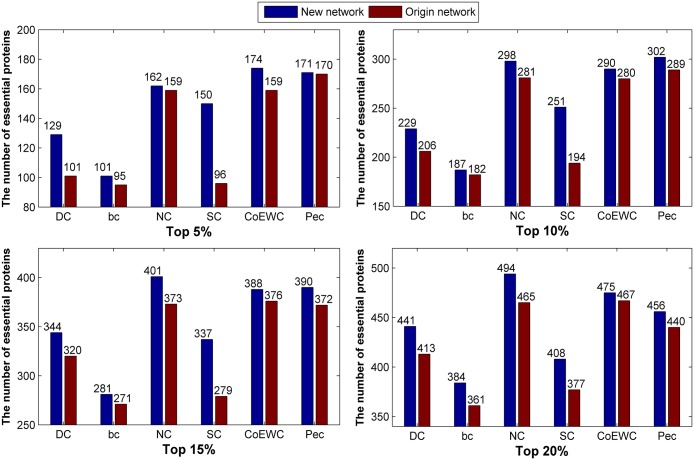
Comparison of the number of essential proteins predicted by the six methods under the new constructed network (network 1) and the original network for 24743_PPI data.

We collect the number of true essential proteins in the top 5%, 10%, 15% and 20% predicted candidate proteins by each method and compare the number of essential proteins identified by six typical methods on the original network and new constructed network (network 1) in Fig 2. As shown in Fig 2, the predicted number of each method under the new constructed network is higher than the original network despite the top 5% ranked proteins.

**Fig 3:**
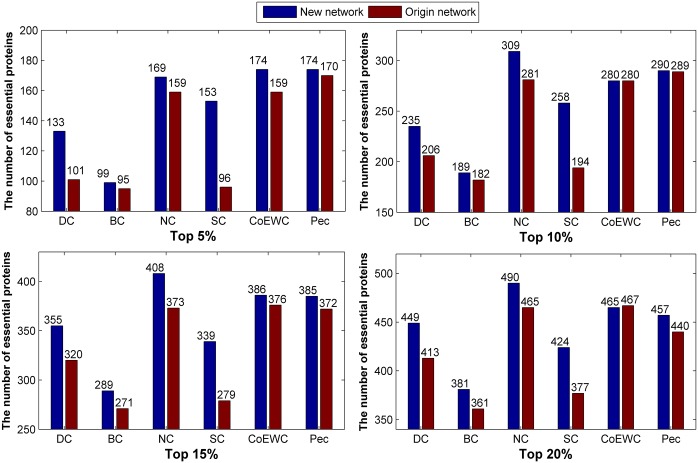
Comparison of the number of essential proteins predicted by the six methods under the new constructed network (network 2) and the original network for 24743_PPI data.

Similarly, we list the number of true essential proteins in the top 5%, 10%, 15% and 20% predicted candidate proteins by each method and compare the number of essential proteins identified by six typical methods on the two networks in Fig 3. Compared to the original network, all of the considered methods achieved comparable or better performance under the new constructed network 2, especially for the topological based methods.

We also validate the efficiency of these methods in predicting essential proteins on the new constructed networks by precision-recall (PR) curve and jackknife curve in the S1 Appendix.

### Analyze the differences of these methods in identifying essential proteins on two networks

The new constructed networks highly improve the performance in predicting essential proteins under the three considered topological-based methods DC, NC, and SC. To further demonstrate the efficiency of the proposed strategy, we analyze the difference between the new constructed network and the original network under the three methods by predicting a small fraction of proteins such as the Top 200. The new network 1 is denoted as new1, the new network 2 is denoted as new2, and the original network is denoted as ori.

The number of overlaps in the top 200 proteins predicted by each method under the new1 and new2 with the original network is denoted as |*ori* ∩ *new*1| and |*ori* ∩ *new*2|, respectively. |*new*1 − *ori*| and |*new*2 − *ori*| denote the number of proteins identified under network 1 and network 2, respectively, but not under the original network for the corresponding methods. Similarly, |*ori* − *new*1| and |*ori* − *new*2| denote the number of proteins identified under the original network but not the new constructed networks for the corresponding methods. The details of essential and non-essential proteins in the intersection and set difference identified by the three methods under the original and new constructed networks for 24734_PPI dataset are summarized in Table 3.

**Table 3:** The number of essential and non-essential proteins in the intersection and set difference identified by three centrality methods under the original network and new constructed networks for 24743_PPI dataset.

			**Number of Essential proteins**			**Number of Non-essential proteins**	
**Methods**	**|*ori* ∩ *new*1|**	**|*new*1 − *ori*|**	**|*ori* ∩ *new*1|**	**|*new*1 − *ori*|**	**|*ori* − *new*1|**	**|*new*1 − *ori*|**	**|*ori* − *new*1|**
DC	139	61	63	42	19	19	42
NC	95	105	55	69	71	36	34
SC	47	153	25	101	52	52	101
			**Number of Essential proteins**			**Number of Non-essential proteins**	
**Methods**	**|*ori* ∩ *new*2|**	**|*new*2 − *ori*|**	**|*ori* ∩ *new*2|**	**|*new*2 − *ori*|**	**|*ori* − *new*2|**	**|*new*2 − *ori*|**	**|*ori* − *new*2|**
DC	147	53	66	44	16	9	37
NC	86	114	49	82	77	32	37
SC	57	143	24	97	53	46	90

The number of essential proteins identified by the three methods under the new constructed networks is relatively larger than the number of essential proteins identified under the original network, especially for the DC and SC methods. For instance, using the DC method, 42 out of 61 proteins are essential in the set difference of new1 and the original network, 44 out of 53 proteins are essential in the set difference of new2 and the original network.

These results show that the new constructed network is more effective than the original network for predicting essential proteins, suggesting that the new predicted links may contribute to the high accuracy in predicting essential proteins.

Similarly, we obtained the number of essential and non-essential proteins in the intersection and set difference identified by three centrality methods under the original network and new constructed networks for the 17201_PPI dataset.

The proportion of added links under the different thresholds of PCC coefficient was shown in S1 Table. To balance the proportion of added links, we also added the GO similarity threshold, and the proportion of new predicted links are listed in the second part of S1 Table when both PCC and GO similarity are satisfied. When the PCC threshold is set to 0.98, the added proportion of links is approximately 19%, and the same proportion of added links is obtained when the PCC threshold is set to 0.96 and the GO similarity threshold is set to 0.4. To test the performance of the new proposed strategy on the 17201_PPI dataset, we set the PCC threshold to 0.98 and obtained the new constructed network as “network 1” (S6 Text) and set the PCC threshold to 0.96 and GO similarity threshold to 0.4 to obtain the new constructed “network 2” (S7 Text) for comparison.

**Fig 4:**
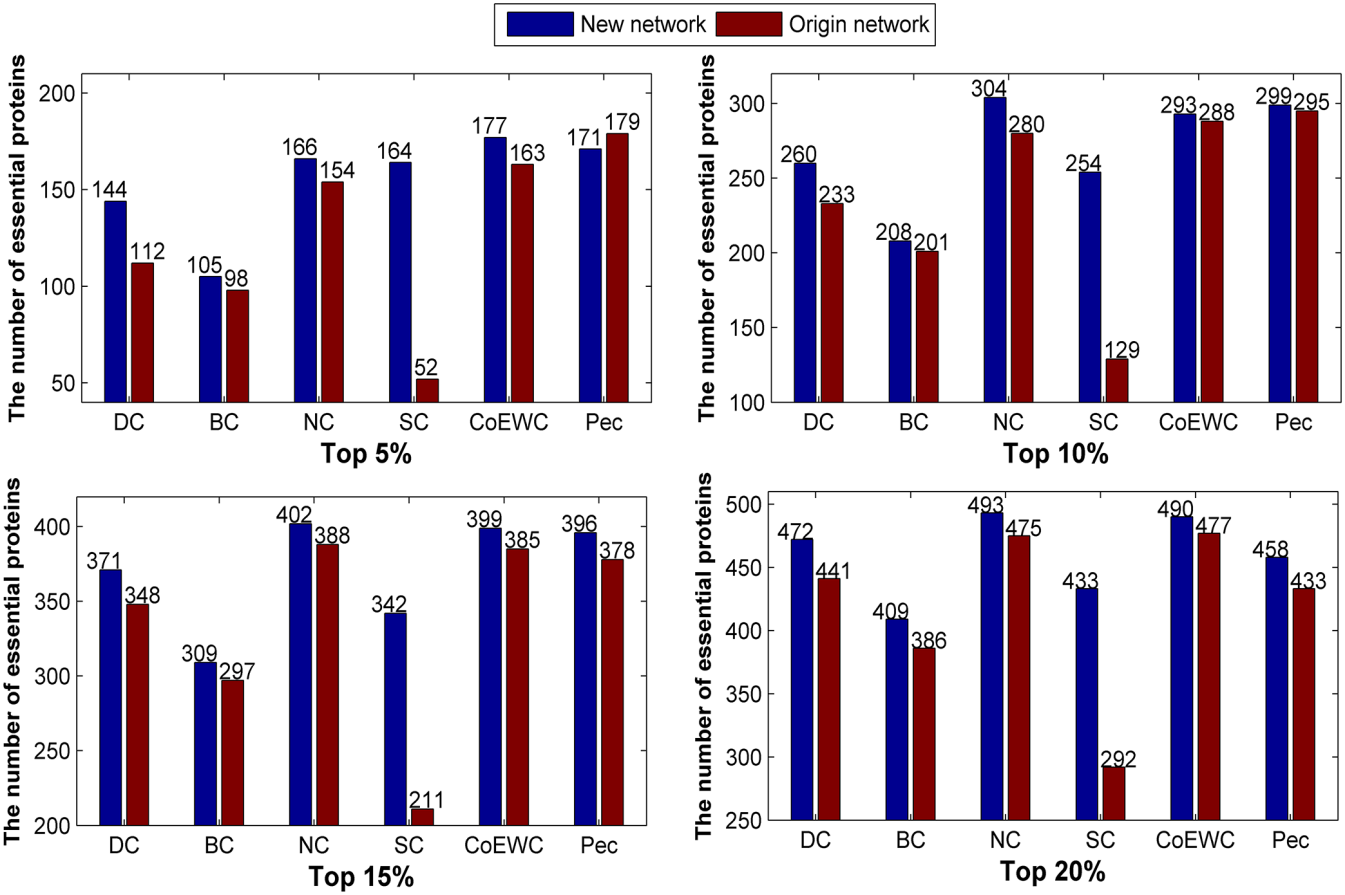
Comparison of the number of essential proteins predicted by the six methods under the new constructed network (“network 1”) and the original network for 17201_PPI data.

**Fig 5:**
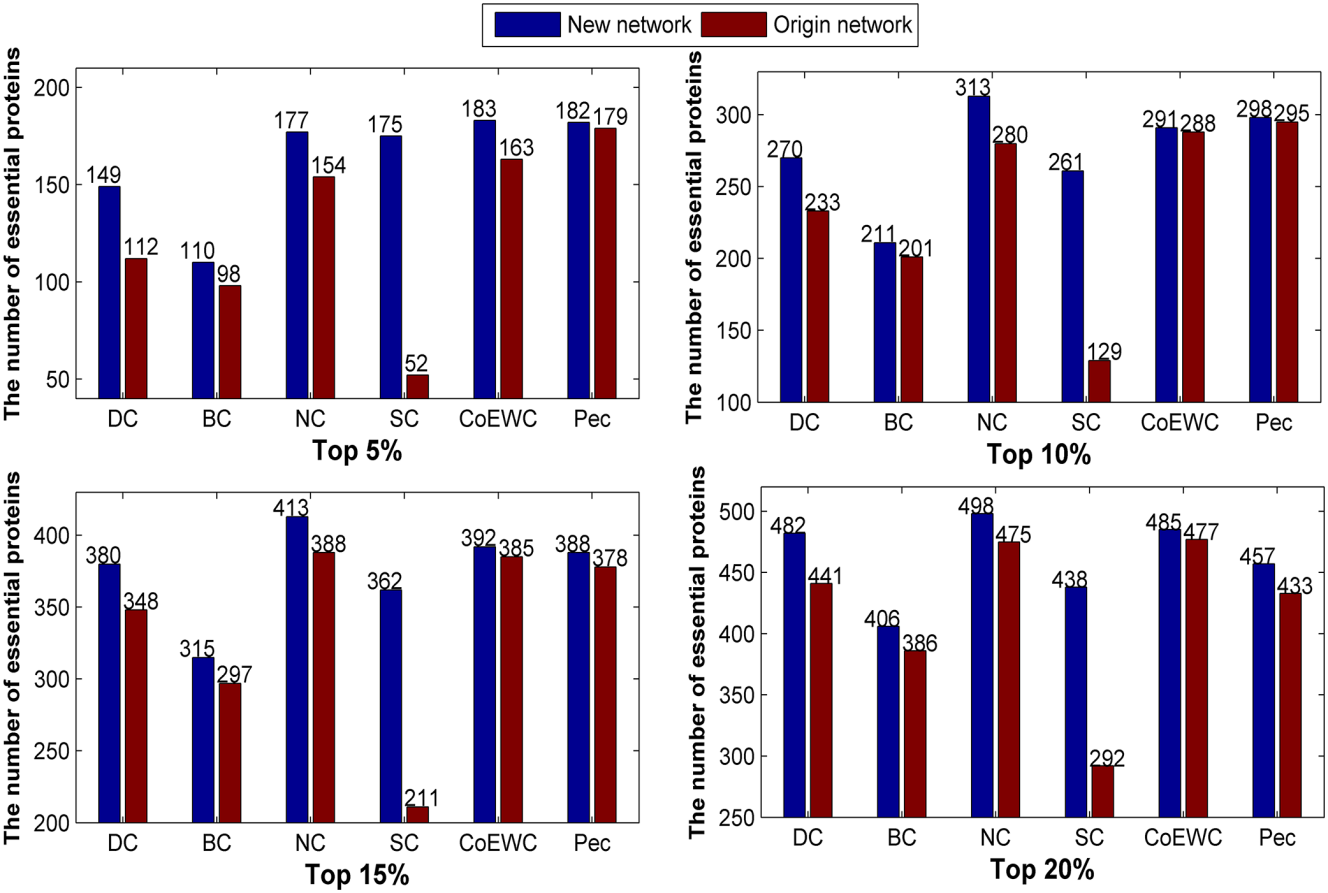
Comparison of the number of essential proteins predicted by the six methods under the new constructed network (“network 2”) and the original network for 17201_PPI data.

We applied the three topological-based methods to the two constructed networks for the 17201_PPI dataset, and the number of true essential proteins in the top 5%, 10%, 15% and 20% predicted candidate proteins by each method are collected and compared with the original network (Figs 4 and 5). Compared to the original network, the new constructed networks show priority in the number of predicted essential proteins under the three methods.

To select the significant essential proteins that could only be predicted under the new constructed networks, we collect the essential and non-essential proteins in the intersection and set difference identified by three centrality methods under the original network and new constructed networks for the 17201_PPI dataset (S2 Table), we can see that the new predicted links are helpful for predicting essential proteins.

For the 14317_PPI dataset, S3 Table demonstrate the proportion of added links under the different thresholds of PCC coefficient and GO similarity. For simplicity, we set the PCC threshold to 0.98 and obtained the new constructed network as “network 1” (S8 Text) and set the PCC threshold to 0.95 and GO similarity threshold to 0.5 to obtain the new constructed “network 2” (S9 Text) for comparison.

**Fig 6:**
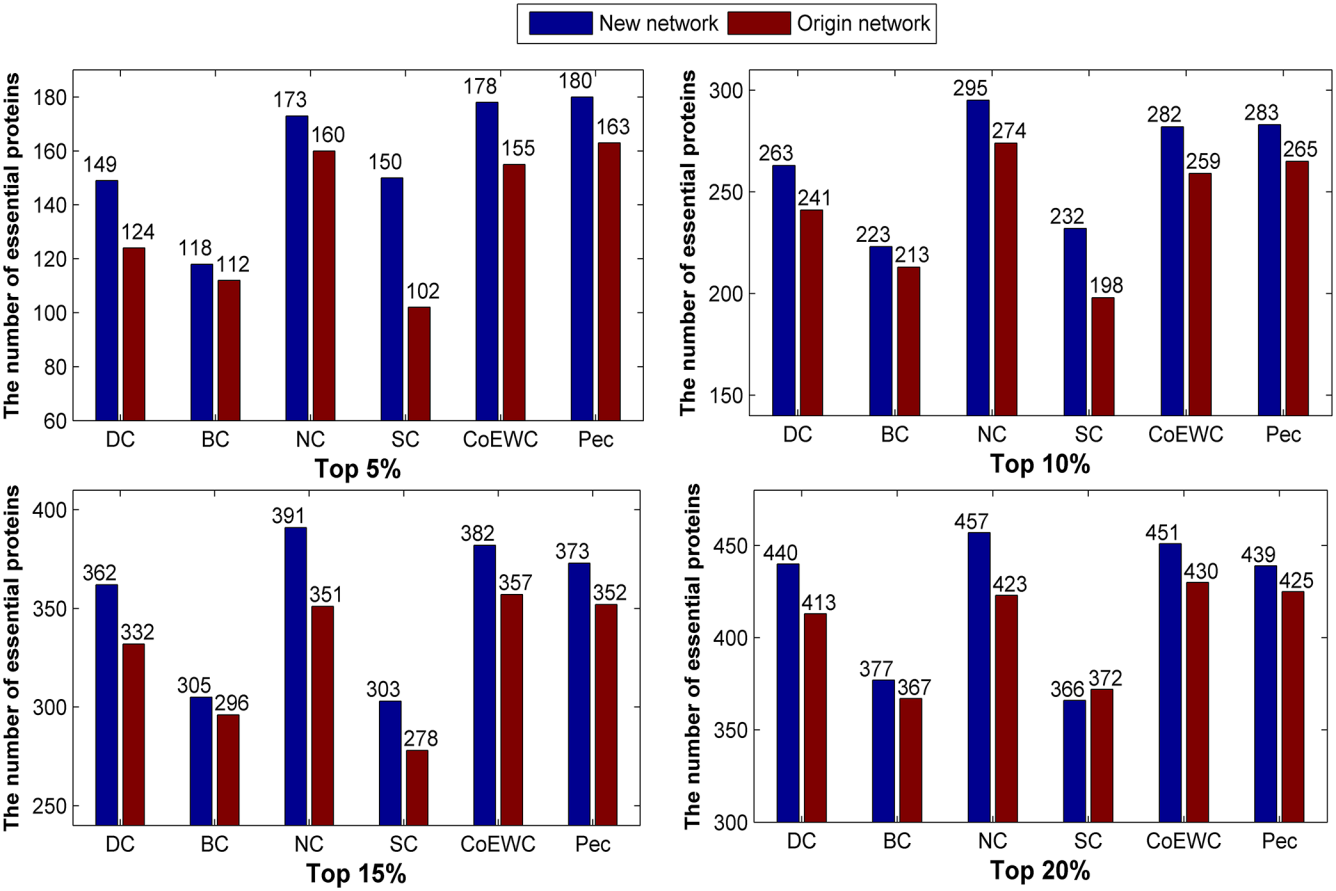
Comparison of the number of essential proteins predicted by the six methods under the new constructed network (“network 1”) and the original network for 14317_PPI data.

**Fig 7:**
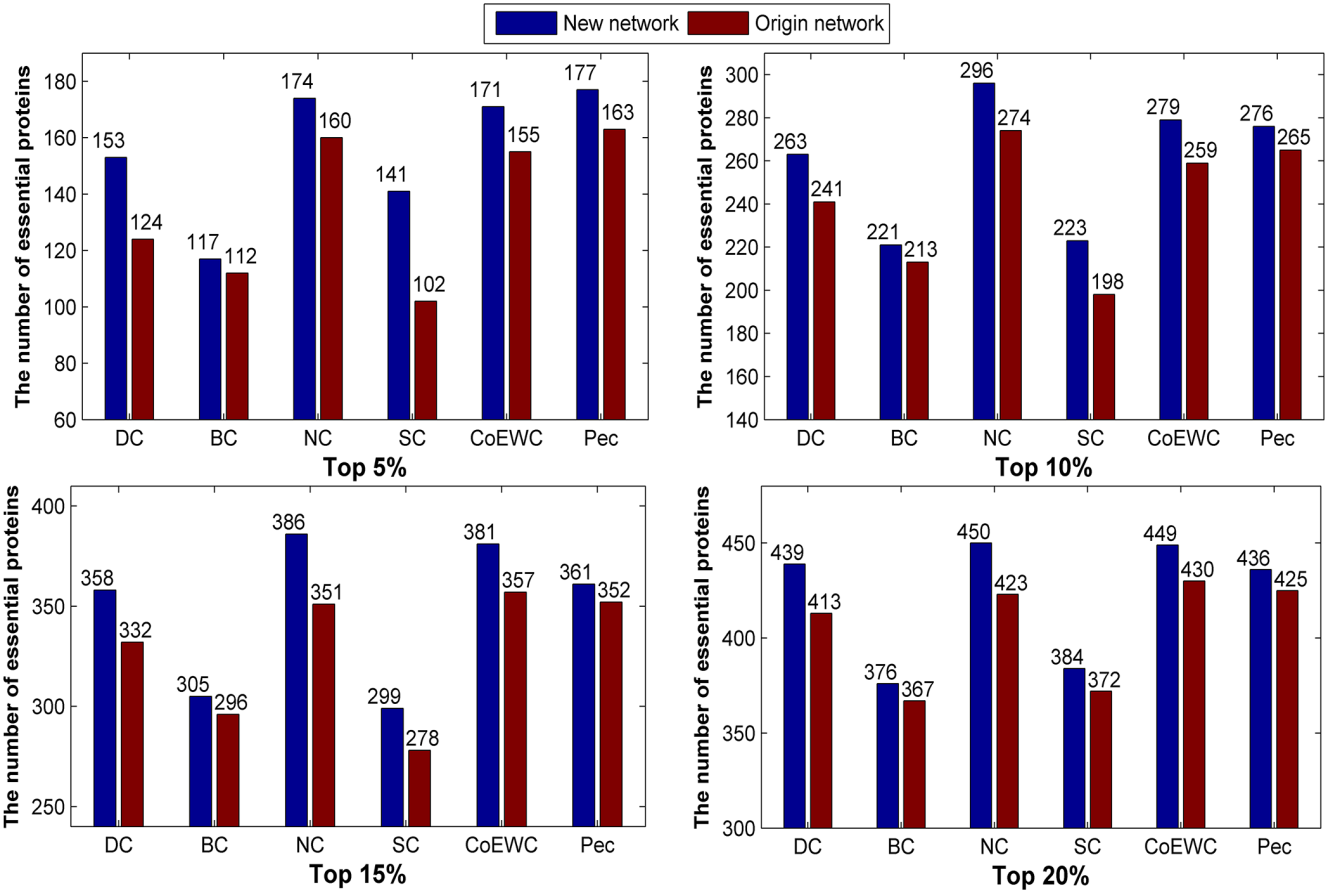
Comparison of the number of essential proteins predicted by the six methods under the new constructed network (“network 2”) and the original network for 14317_PPI data.

Similarly, we applied the three topological-based methods to the two constructed networks for the 14317_PPI dataset, and the number of true essential proteins in the top 5%, 10%, 15% and 20% predicted candidate proteins by each method are collected and compared with the original network (Figs 6 and 7). Compared to the original network, the new constructed networks show priority in the number of predicted essential proteins under the three methods.

To select the significant essential proteins that could only be predicted under the new constructed networks, we collect the essential and non-essential proteins in the intersection and set difference identified by three centrality methods under the original network and new constructed networks for the 14317_PPI dataset (S4 Table).

### Validate the new predicted links

To reveal the contribution of the new predicted links on high prediction rates for the three topological-based centrality methods, the following conditions are set. First, we select the candidate proteins that could only be predicted by the three methods in the top 200 under the two new constructed networks for the 24743_PPI dataset, 17201_PPI dataset and 14317_PPI dataset., and then we validate the essentiality of the candidate proteins by true benchmark essential proteins set, at last, the true essential proteins in the candidate proteins are selected.

For the 24743_PPI dataset, there are 21 proteins that satisfy the condition under the two new constructed networks. To obtain an overview of these 21 proteins in the original network, we calculate the three centrality measures and sort their value in a descending order. The protein name and corresponding rank position under the three different methods in the original network are listed in Table 4.

**Table 4:** The rank position of the selected proteins under the original network by the three corresponding methods for the 24743_PPI data.

Protein name	rank in SC	rank in DC	rank in NC
YCL054W	1212	1338	938
YDL060W	959	715	556
YDR087C	887	1067	666
**YGR159C**	**1363**	**2151**	**3369**
YJL069C	385	288	304
YLR186W	260	694	1572
YLR222C	237	386	962
**YLR276C**	**1782**	**1749**	**1247**
**YML093W**	**3703**	**4786**	**4280**
**YMR128W**	**2007**	**1301**	**1207**
**YMR131C**	**3960**	**3837**	**4354**
YMR290C	308	391	231
**YNL062C**	**3845**	**3871**	**1121**
**YNL075W**	**2468**	**2645**	**1122**
**YNL112W**	**2200**	**1781**	**2295**
YNL308C	709	910	1870
**YNR054C**	**2887**	**3912**	**1128**
**YOR004W**	**1854**	**3939**	**4708**
YOR272W	291	395	219
YPL012W	672	663	990
YPR144C	2101	2340	874

As shown in Table 4, 21 true essential proteins are ranked in the top 200 under the new constructed networks, but almost half of these proteins (10 out of 21) ranked over 1000 in the original network. This finding demonstrates that the new predicted links associated with these proteins may be statistically significant, and their existence is highly probable.

To validate the new predicted links, we first select the 10 proteins collected in Table 4 and collect the new predicted interactions associated with the 10 proteins in both of the new constructed networks. Then, we collect the interactions associated with these proteins by mining the STRING database (STRING Database. http://string.embl.de/) and filtering the interactions with a confidence score smaller than 0.7. The overlaps between the new predicted interactions and interactions collected in the STRING database are assumed to be truly predicted. For comparison, the total new predicted links in the new constructed networks, i.e., network 1 and network 2, are also validated by using the reliable links obtained from the STRING database.

Table 5 shows the number and fraction of edges in different groups validated by the STRING database. The precision of the selected predicted links associated with the 10 selected proteins is higher than the total new predicted links under both new constructed networks.

**Table 5:** Validation of the total new predicted links and the new predicted links associated with the 10 proteins by STRING database for the 24743_PPI dataset.

Edge group	Number of edges	
	New network 1	New network 2
Total predicted	3321	3326
Confirmed	2343(0.706)	2098(0.631)
Select predicted	653	609
Confirmed	521(0.798)	467(0.767)

Values in parentheses are fraction of edges validated.

The total new predicted interactions in the two new constructed networks and validated interactions, the new predicted interactions associated with the 10 proteins under the two new constructed networks, and the confirmed interactions under 24743_PPI dataset were presented in S1 File at the supplemental part.

**Fig 8:**
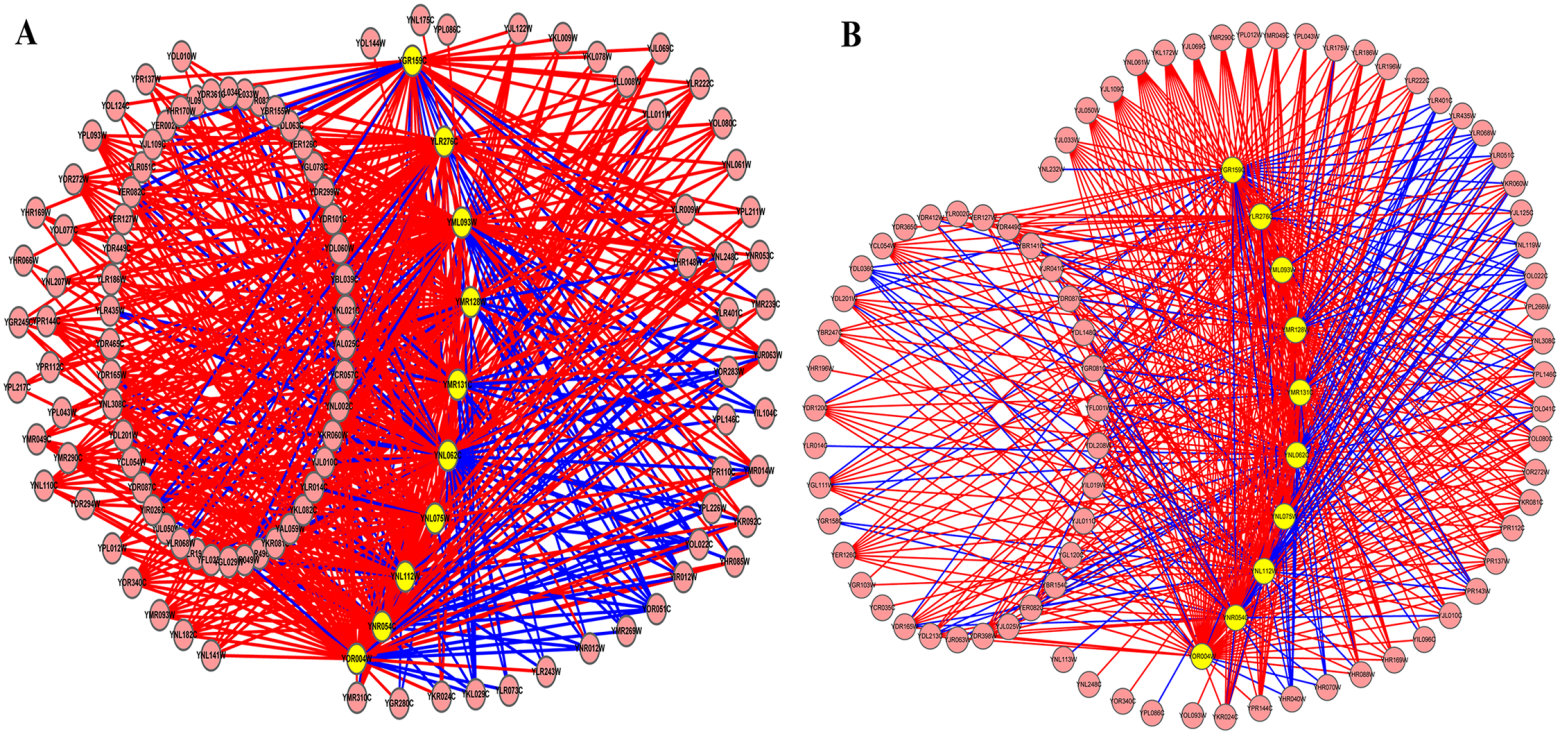
The new predicted links associated with 10 proteins (marked yellow) under the two new constructed networks. The edges that have been validated by the interactions collected in the STRING database are marked in red, and the other edges are marked in green. (A) The new predicted edges associated with 10 proteins in the new constructed network 1. (B) The new predicted edges associated with 10 proteins in the new constructed network 2.

The true predicted interactions associated with the 10 proteins under the two new constructed networks are displayed in Fig 8. For the new constructed network 1521 edges (involving 108 proteins) out of 653 edges (79.8%) are validated, and for the network 2467 edges (involved 75 proteins) out of 609 edges (76.7%) are validated by the STRING database.

For the 17201_PPI dataset, there are 37 proteins satisfying the condition under both new constructed networks. Similarly, we calculate the three centrality measures and sort their value in descending order. The protein name and corresponding rank position under three different methods in the original network are listed in Table 6.

**Table 6:** The rank position of the selected proteins under the original network by the three corresponding methods for the 17201_PPI data.

Protein name	rank in SC	rank in DC	rank in NC
**YCL054W**	**1847**	**1111**	**1452**
YDL060W	1593	511	538
YDR087C	453	1297	1832
YDR449C	2117	1306	834
YER126C	749	347	251
**YGR159C**	**3567**	**2601**	**4523**
YGR245C	1023	437	306
**YHR148W**	**2496**	**1342**	**1014**
YHR169W	1608	477	1064
**YIL091C**	**4489**	**4282**	**2511**
YJL033W	1475	395	420
**YJL050W**	**3691**	**2181**	**4793**
YLL011W	1770	703	1450
YLR009W	718	2221	4804
YLR186W	2226	1055	898
YLR196W	527	244	838
YLR222C	903	331	467
YLR276C	1884	1383	814
**YML093W**	**4113**	**4585**	**2830**
YMR128W	1774	944	1292
**YMR131C**	**4810**	**4620**	**2868**
YMR290C	340	248	388
YNL002C	1225	852	407
**YNL062C**	**3145**	**3599**	**1711**
**YNL075W**	**2104**	**2304**	**1426**
**YNL112W**	**2088**	**1906**	**3601**
YNL308C	402	604	3497
**YNR054C**	**3431**	**3642**	**1722**
**YOL022C**	**2731**	**4733**	**2995**
**YOR004W**	**3325**	**4764**	**3027**
YOR272W	606	309	255
YPL012W	1051	610	1148
**YPL217C**	**2085**	**1962**	**1785**
**YPR112C**	**3389**	**3774**	**4316**
**YPR137W**	**2890**	**1439**	**1289**
**YPR144C**	**2209**	**1970**	**1237**

As shown in Table 6, 37 true essential proteins are ranked in the top 200 under the new constructed networks, but 17 out of these ranked over 1000 in the original network. To validate the new predicted links, we first select the 17 proteins collected in Table 6 and collect the new predicted interactions associated with the 17 proteins in both constructed networks. Similarly, we validate the new predicted interactions associated with the 17 proteins by using the reliable interactions obtained from the STRING database.

Table 7 shows the number and fraction of edges in different groups validated by the STRING database. The precision of the selected predicted links associated with the 17 selected proteins is higher than the total new predicted links under both new constructed networks, i.e. “network 1” and “network 2”.

**Table 7:** Validation of the total new predicted links and the new predicted links associated with the 17 proteins by STRING database for the 17201_PPI data.

Edge group	Number of edges	
	New network 1	New network 2
Total predicted	3265	3269
Confirmed	2344(0.718)	2066(0.632)
Select predicted	957	948
Confirmed	759(0.793)	697(0.735)

Values in parentheses are fraction of edges validated.

The total new predicted interactions in the two new constructed networks and validated interactions, the new predicted interactions associated with the 17 proteins under the two new constructed networks, and the confirmed interactions under 17201_PPI dataset were shown in S1 File at the supplemental part.

For the 14317_PPI dataset, there are 24 proteins satisfying the condition under both new constructed networks. Similarly, we calculate the three centrality measures and sort their value in descending order. The protein name and corresponding rank position under three different methods in the original network are listed in Table 8.

**Table 8:** The rank position of the selected proteins under the original network by the three corresponding methods for the 14317_PPI data.

Protein name	rank in SC	rank in DC	rank in NC
YCL054W	720	1336	1403
YDR449C	348	620	667
**YER082C**	**1770**	**2230**	**2171**
**YER126C**	**2128**	**2232**	**2184**
YIL091C	675	1110	1520
YJL069C	842	1118	537
**YLR186W**	**1507**	**2454**	**2937**
YLR196W	215	358	646
YLR222C	1512	847	458
YLR276C	541	1446	838
YML093W	879	702	1510
**YMR131C**	**2414**	**1688**	**3118**
**YNL062C**	**1533**	**2000**	**3219**
**YNL075W**	**2349**	**2536**	**3222**
**YNL112W**	**868**	**1140**	**1192**
YNR054C	282	338	424
**YOL022C**	**2268**	**3500**	**3337**
**YOR004W**	**1935**	**3526**	**3391**
YOR272W	460	1299	556
YPL012W	504	601	1588
YPL217C	539	782	1301
**YPR112C**	**1493**	**1160**	**3644**
YPR137W	598	1059	889
YPR144C	848	664	1308

Due to the number of true essential proteins in 14317_PPI dataset is less than 1000, here we set the rank threshold to 800. As shown in Table 8, 24 true essential proteins are ranked in the top 200 under the new constructed networks, but 10 out of these ranked over 800 in the original network. To validate the new predicted links, we first select the 10 proteins collected in Table 8 and collect the new predicted interactions associated with the 10 proteins in both constructed networks. Similarly, we validate the new predicted interactions associated with the 10 proteins by using the reliable interactions obtained from the STRING database.

Table 9 shows the number and fraction of edges in different groups validated by the STRING database. The precision of the selected predicted links associated with the 10 selected proteins is higher than the total new predicted links under both new constructed networks, i.e. “network 1” and “network 2”.

**Table 9:** Validation of the total new predicted links and the new predicted links associated with the 10 proteins by STRING database for the 14317_PPI data.

Edge group	Number of edges	
	New network 1	New network 2
Total predicted	2598	2662
Confirmed	1951(0.751)	1739(0.653)
Select predicted	530	561
Confirmed	421(0.794)	407(0.725)

Values in parentheses are fraction of edges validated.

The total new predicted interactions in the two new constructed networks and validated interactions, the new predicted interactions associated with the 10 proteins under the two new constructed networks, and the confirmed interactions under 14317_PPI dataset were presented in S3 File at the supplemental part.

To compare the performance of the new method with the four other state-of-the-art link prediction methods in predicting underlying links in the PPI network, we applied the three methods on the test PPI network and selected the top ranked edges (approximately 13% of the edges for the original network of 24743_PPI dataset and approximately 19% of the edges for the original network of 17201_PPI dataset, and approximately 18% of the edges for the original network of 14317_PPI dataset) as a new predicted interaction between unlinked protein pairs. The new predicted links are validated by mining the STRING database with a high confidence score. The number of new predicted links and confirmed links for each method are listed in Table 10. Compared with the results list in Tables 5, 7 and 9 we can see that the prediction accuracy of the new proposed method is much better than these random walk-based methods.

**Table 10:** The Number of predicted links and confirmed links for the four random walk-based methods.

Methods	24743_PPI		17201_PPI		14317_PPI	
	Predicted	Confirmed	Predicted	Confirmed	Predicted	Confirmed
RWS	3217	270(0.0839)	3268	367(0.112)	2577	271(0.105)
LRW	3226	231(0.0716)	3269	252(0.0771)	2578	248(0.096)
SRW	3222	238(0.0739)	3279	285(0.0869)	2579	227(0.088)
RWR	3218	271(0.0842)	3269	290(0.0887)	2578	249(0.096)

Values in parentheses are fraction of edges validated.

## Conclusions

Predicting interactions between two proteins is a hot topic in the post-genome era, although many computational methods have been proposed to predict links for the complex network. However, most of these methods are topological-based, and the accuracy of these methods remains unsatisfied.

The two linked proteins are more likely to be co-expressed and have the similar biological function. In the present work, we propose a new strategy to predict underlying links between two originally unlinked proteins based on two-stages. We first construct new networks by measuring the co-expressed score and GO similarity score of unlinked protein pairs and then select the significant part of new predicted interactions by comparing the essential proteins identified using the new constructed networks and original network.

To evaluate the performance of the new strategy, we validate the new predicted links using the high confidence interactions obtained from the STRING database. Simulation results show that the prediction accuracy can be highly improved under the new proposed strategy for both test datasets.

Our method may be improved in two directions. On the one hand, it provides new insight for computationally predicting new interactions through measuring the co-expression and GO functional similarity of unlinked protein pairs, and it provides new insight for selecting the significant part of new predicted interactions by mining the difference between the new constructed network and original network for identifying essential proteins. On the other hand, we can increase the accuracy of prediction essential proteins by improving the integrity of PPI.

Although the new strategy performs well in the detection of underlying links in the PPI network, the network obtained by link prediction is still rough, and the false-positive and negative links in the networks have not been considered. Therefore, in the future, we will work to design refined measures in predicting unrevealed links between protein pairs by reasonable integration of PPI topological information with other types of high throughput data, and we will work to filter noise underlying the PPI network.

## Supporting information

S1 Text Protein interaction data of original network with 5093 proteins and 24743 interactions. (TXT)

S2 Text Protein interaction data of original network with 4928 proteins and 17201 interactions. (TXT)

S3 Text Protein interaction data of original network with 3672 proteins and 14317 interactions. (TXT)

S4 Text Protein interaction data of new constructed network 1 under 24743_PPI dataset. (TXT)

S5 Text Protein interaction data of new constructed network 2 under 24743_PPI dataset. (TXT)

S6 Text Protein interaction data of new constructed network 1 under 17201_PPI dataset. (TXT)

S7 Text Protein interaction data of new constructed network 2 under 17201_PPI dataset. (TXT)

S8 Text Protein interaction data of new constructed network 1 under 14317_PPI dataset. (TXT)

S9 Text Protein interaction data of new constructed network 2 under 14317_PPI dataset. (TXT)

S1 Table The proportion of added links under different PCC threshold for the 17201_PPI dataset. (DOCX)

S2 Table The number of essential and non-essential proteins in the intersection and set difference identified by the three centrality methods under the original network and the new constructed networks for predicting the top 200 proteins under the 17201_PPI dataset. (DOCX)

S3 Table The proportion of added links under different PCC and GO similarity thresholds for the 14317_PPI dataset. (DOCX)

S4 Table The number of essential and non-essential proteins in the intersection and set difference identified by the three centrality methods under the original network and the new constructed networks for predicting the top 200 proteins under the 14317_PPI dataset. (DOCX)

S1 Appendix The definition of the six centrality measures and comparison of the efficiency these methods in predicting essential proteins on considered networks. (DOCX)

S1 Fig Comparing the performance of the six centrality measures on the original network and new constructed network 1 by RP curve for the 24743_PPI dataset. (TIF)

S2 Fig Comparing the performance of the six centrality measures for the original network and new constructed network 2 by the RP curve for the 24743_PPI dataset. (TIF)

S3 Fig The performances of the six centrality measures for the original network and new constructed network 1 using a jackknife methodology under the 24743_PPI dataset. (TIF)

S4 Fig The performances of the six centrality measures for the original network and new constructed network 2 using a jackknife methodology under the 24743_PPI dataset. (TIF)

S5 Fig Comparing the performance of the six centrality measures for the original network and new constructed network 1 using the RP curve for the 17201_PPI dataset. (TIF)

S6 Fig Comparing the performance of the six centrality measures for the original network and new constructed network 2 using the RP curve for the 17201_PPI dataset. (TIF)

S7 Fig Comparing the performance of the six centrality measures for the original network and new constructed network 1 using the RP curve for the 14317_PPI dataset. (TIF)

S8 Fig Comparing the performance of the six centrality measures for the original network and new constructed network 2 using the RP curve for the 14317_PPI dataset. (TIF)

S1 File The file contains the total of new predicted interactions in the two new constructed networks and confirmed interactions, the selected significant part of new predicted interactions associated with the 10 proteins under the two new constructed networks and the confirmed interactions for 24743_PPI dataset. (XLSX)

S2 File The file contains the total of new predicted interactions in the two new constructed networks and confirmed interactions, the selected significant part of new predicted interactions associated with the 17 proteins under the two new constructed networks and the confirmed interactions for 17201_PPI dataset. (XLSX)

S3 File The file contains the total of new predicted interactions in the two new constructed networks and confirmed interactions, the selected significant part of new predicted interactions associated with the 10 proteins under the two new constructed networks and the confirmed interactions for 14317_PPI dataset. (XLSX)
