# Supplementary material for: A new two-stage method for revealing missing parts of edges in protein-protein interaction networks
Source: PLoS One. 2017 May 11;12(5):e0177029. doi: 10.1371/journal.pone.0177029 (PMC5426645; doi:10.1371/journal.pone.0177029)
Supplement: S1 Table — (DOCX) [file pone.0177029.s010.docx]

**Table S1. The proportion of added links under different PCC threshold for the 17201_PPI dataset.**

| PCC | 0.95 | 0.96 | 0.97 | 0.98 | 0.99 |
| --- | --- | --- | --- | --- | --- |
| Added Proportion | 1.144 | 0.771 | 0.450 | 0.190 | 0.027 |
| PCC & GO_sim | 0.95 & 0.5 | 0.96 & 0.4 | 0.97 & 0.3 | 0.98 & 0.2 | 0.99 & 0.1 |
| Added Proportion | 0.192 | 0.190 | 0.193 | 0.085 | 0.012 |
