# Supplementary material for: A new two-stage method for revealing missing parts of edges in protein-protein interaction networks
Source: PLoS One. 2017 May 11;12(5):e0177029. doi: 10.1371/journal.pone.0177029 (PMC5426645; doi:10.1371/journal.pone.0177029)
Supplement: S2 Table — (DOCX) [file pone.0177029.s011.docx]

**Table S2. The number of essential and non-essential proteins in the intersection and set difference identified by the three centrality methods under the original network and the new constructed networks for predicting the top 200 proteins under the 17201_PPI dataset.**

| Methods | \|ori∩new1\| | \|new1-ori\| | Number of Essential proteins | | | Number of Non-essential proteins | |
| --- | --- | --- | --- | --- | --- | --- | --- |
|  |  |  | \|ori∩new1\| | \|new1-ori\| | \|ori-new1\| | \|new1-ori\| | \|ori-new1\| |
| DC | 112 | 88 | 58 | 57 | 33 | 31 | 55 |
| NC | 79 | 121 | 46 | 82 | 50 | 71 | 39 |
| SC | 4 | 196 | 2 | 134 | 40 | 52 | 156 |
| Methods | \|ori∩new1\| | \|new1-ori\| | Number of Essential proteins | | | Number of Non-essential proteins | |
|  |  |  | \|ori∩new1\| | \|new1-ori\| | \|ori-new1\| | \|new1-ori\| | \|ori-new1\| |
| DC | 68 | 132 | 43 | 88 | 48 | 44 | 84 |
| NC | 105 | 95 | 61 | 72 | 35 | 23 | 60 |
| SC | 5 | 195 | 3 | 140 | 39 | 55 | 156 |
