# Supplementary material for: A new two-stage method for revealing missing parts of edges in protein-protein interaction networks
Source: PLoS One. 2017 May 11;12(5):e0177029. doi: 10.1371/journal.pone.0177029 (PMC5426645; doi:10.1371/journal.pone.0177029)
Supplement: S3 Table — (DOCX) [file pone.0177029.s012.docx]

**Table S3. The proportion of added links under different PCC and GO similarity thresholds for the 14317_PPI dataset.**

| PCC | 0.95 | 0.96 | 0.97 | 0.98 | 0.99 |
| --- | --- | --- | --- | --- | --- |
| Added Proportion | 1.028 | 0.714 | 0.429 | 0.181 | 0.026 |
| PCC & GO_sim | 0.95 & 0.5 | 0.96 & 0.4 | 0.97 & 0.3 | 0.98 & 0.2 | 0.99 & 0.1 |
| Added Proportion | 0.186 | 0.192 | 0.183 | 0.1016 | 0.019 |
