# Supplementary material for: A new two-stage method for revealing missing parts of edges in protein-protein interaction networks
Source: PLoS One. 2017 May 11;12(5):e0177029. doi: 10.1371/journal.pone.0177029 (PMC5426645; doi:10.1371/journal.pone.0177029)
Supplement: S1 Appendix — (DOCX) [file pone.0177029.s014.docx]

**A1.** **The definition of centrality measures**

1、The Degree Centrality (DC) of a vertex u is defined as the degree of node u.

2、The Betweenness Centrality (BC) of a vertex u is defined as the fracStion of shortest paths that pass through the node u.

$BC(u)=\sum_{s\neq u\neq t} \frac{\sigma_{st}(u)}{\sigma_{st}}$ (1)

where $\sigma_{st}$ denotes the number of shortest paths from s to t, and $\sigma_{st}(u)$ is the number of shortest paths from s to t on which u lies.

3、The Subgraph Centrality (SC) of a node u is denoted as the total number of closed walks in which v takes part and gives more weight to closed walks of short lengths.

$SC(u)=\sum_{k=0}^{\infty} \frac{\mu_{k}(u)}{l!}=\sum_{j=1}^{N} [v_{j}^{u}]^{2}e^{\lambda_{j}}$ (2)

where$\mu_{k}(u)$is the number of closed walks of length$l$starting and ending at protein$u$;$v_{1},v_{2},\cdots v_{N}$ is an orthonormal basis of$R_{N}$composed by eigenvectors of the adjacency matrix A of the network and $\lambda_{1},\lambda_{2},\cdots,\lambda_{N}$ are the corresponding eigenvalues; and$v_{j}^{u}$denotes the u-th component of$v_{j}$.

4、For a protein u, the essentiality $NC(u)$is defined as the sum of edge clustering coefficients ($ECC$) of all edges directly connected with node u.

$NC(u)=\sum_{v\in N(u)} ECC(u,v)=\sum_{v\in N(u)} \frac{N_{u,v}^{3}}{\min(d_{u}-1,d_{v}-1)}$ (3)

where $d_{u}$and $d_{v}$are the degrees of node u and v, respectively. $N_{u,v}^{3}$ denotes the number of triangles composed of the edge (u, v) in the network, and$N_{u}$denotes the set of all neighbors of node u.

5、For a protein u, the essentiality $Pec(u)$ is defined as

$Pec(u)=\sum_{v\in N(u)} \frac{|N_{u}\cap N_{v}|+1}{\min\{d_{u},d_{v}\}}\times PCC(u,v)$ (4)

where $N_{u}$(or $N_{v}$) is the set of neighbors of vertex *u* (or *v*), and $d_{u}$(or $d_{v}$) denotes the degree of vertex *u* (or *v*) .

6、For a protein u, the essentiality $CoEWC(u)$ is defined as the sum of the PCC between u and each of its neighbors weighted by the corresponding neighbor’s clustering coefficient.

$CoEWC(u)=\sum_{v\in N(u)} C(v)\times PCC(u,v)$ (5)

where$N_{u}$denotes the set of all neighbors of node u, and $C(v)$ denotes the clustering coefficient of node v.

**A2 Validated by other evaluation measures**

To evaluate the performance of the predicted precision of these methods on the considered networks, we plot the precision-recall (PR) curve of these methods on the new constructed networks and the original network. The precision and recall of the top n ranked proteins are defined as follows

$\Pr ecision(n)=\frac{TP(n)}{TP(n)+FP(n)}$ (6)

$\mathrm{Re}call(n)=\frac{TP(n)}{P}$ (7)

where $TP(n)$is the number of true predicted essential proteins among the top n ranked proteins. $FP(n)$ is the number of false predicted essential proteins among the top n ranked proteins.

We compared the considered methods for the original network and the two constructed networks by plotting precision as a function of recall in S1 and S2 Figs. We can see that the PR curves of the considered topological-based methods such as DC, NC, and SC show a significant improvement under the new constructed networks, but for the Pec, the PR curves under the new constructed networks perform comparably and slightly better than the original networks. For the CoEWC, the precision of the new constructed networks is higher than the original networks when recall is larger than 0.1.

To further validate the performance of the new proposed strategy, we adapt the jackknife methodology [1] and plot the cumulative count of essential proteins with respect to the number of top ranked proteins for different methods under the two networks. The subfigures in S3 Fig show the comparison result on the new constructed network 1. The jackknife curve of these methods on the new network is higher than that of the original network, especially for the topological-based methods such as DC, NC and SC.

The jackknife curve of these methods on the new constructed network 2 can be observed in S4 Fig. The topological-based methods always achieved comparable or better performance under the new constructed network compared to the original network.

Similarly, we compare the PR curves for the original networks and new constructed networks under 17201_PPI dataset (S5 and S6 Figs) and 14317_PPI dataset (S7 and S8 Figs). The PR curves of the considered topological-based methods such as DC, NC, and SC show a significant improvement under the new constructed networks, but for the Pec and CoEWC, the PR curves under the new constructed networks perform comparably and slightly better than the original networks.

**References:**

[1] Holman AG, Davis PJ, Foster JM, Carlow CKS, Kumar S. Computational prediction of essential genes in an unculturable endosymbiotic bacterium, Wolbachia of Brugia malayi. BMC Microbiology. 2009 Nov; 9(1): 243.
