# Supplementary figures and images for: A new two-stage method for revealing missing parts of edges in protein-protein interaction networks
Source: PLoS One. 2017 May 11;12(5):e0177029. doi: 10.1371/journal.pone.0177029 (PMC5426645; doi:10.1371/journal.pone.0177029)

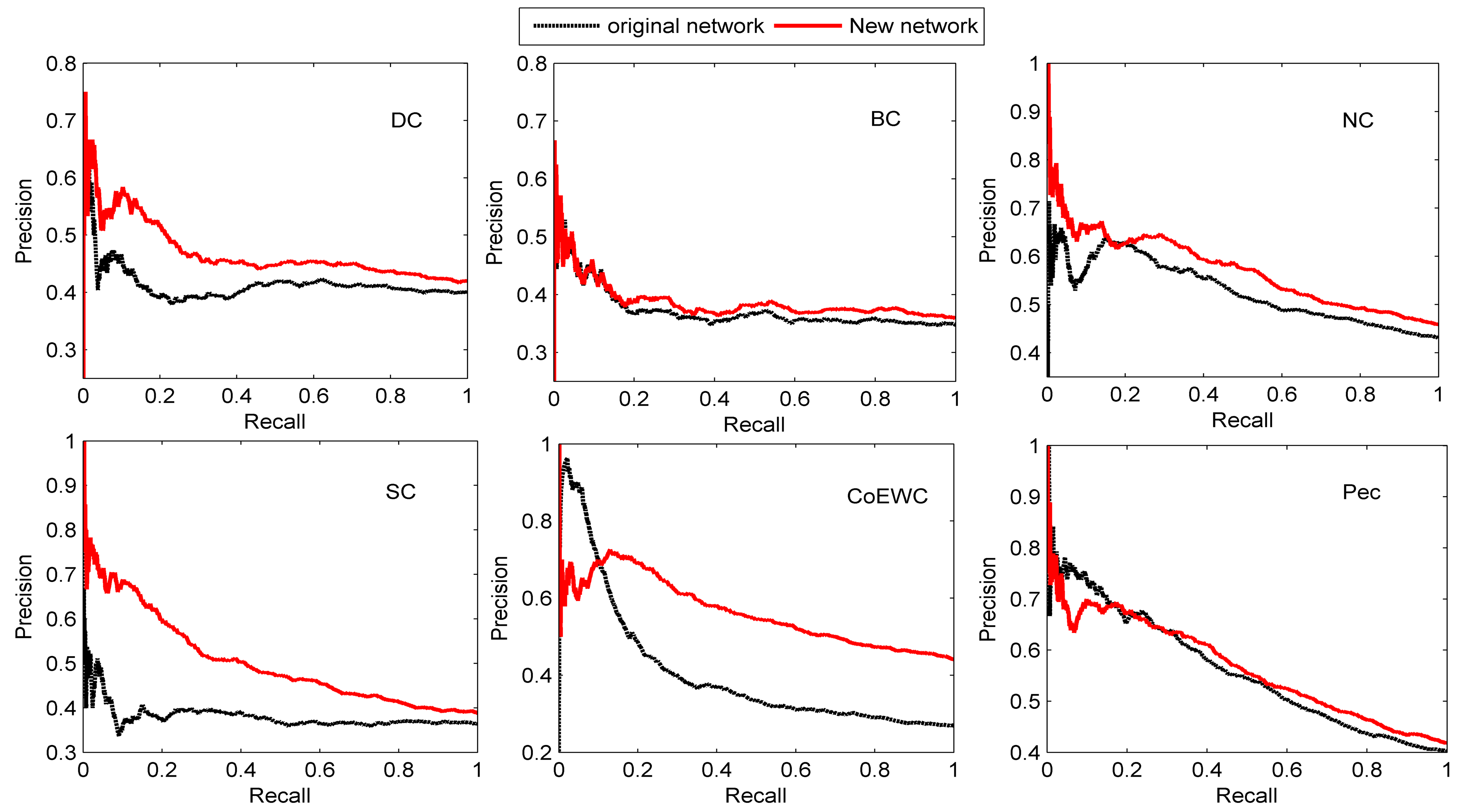

Supplement: S1 Fig — (TIF) [file pone.0177029.s015.tif]

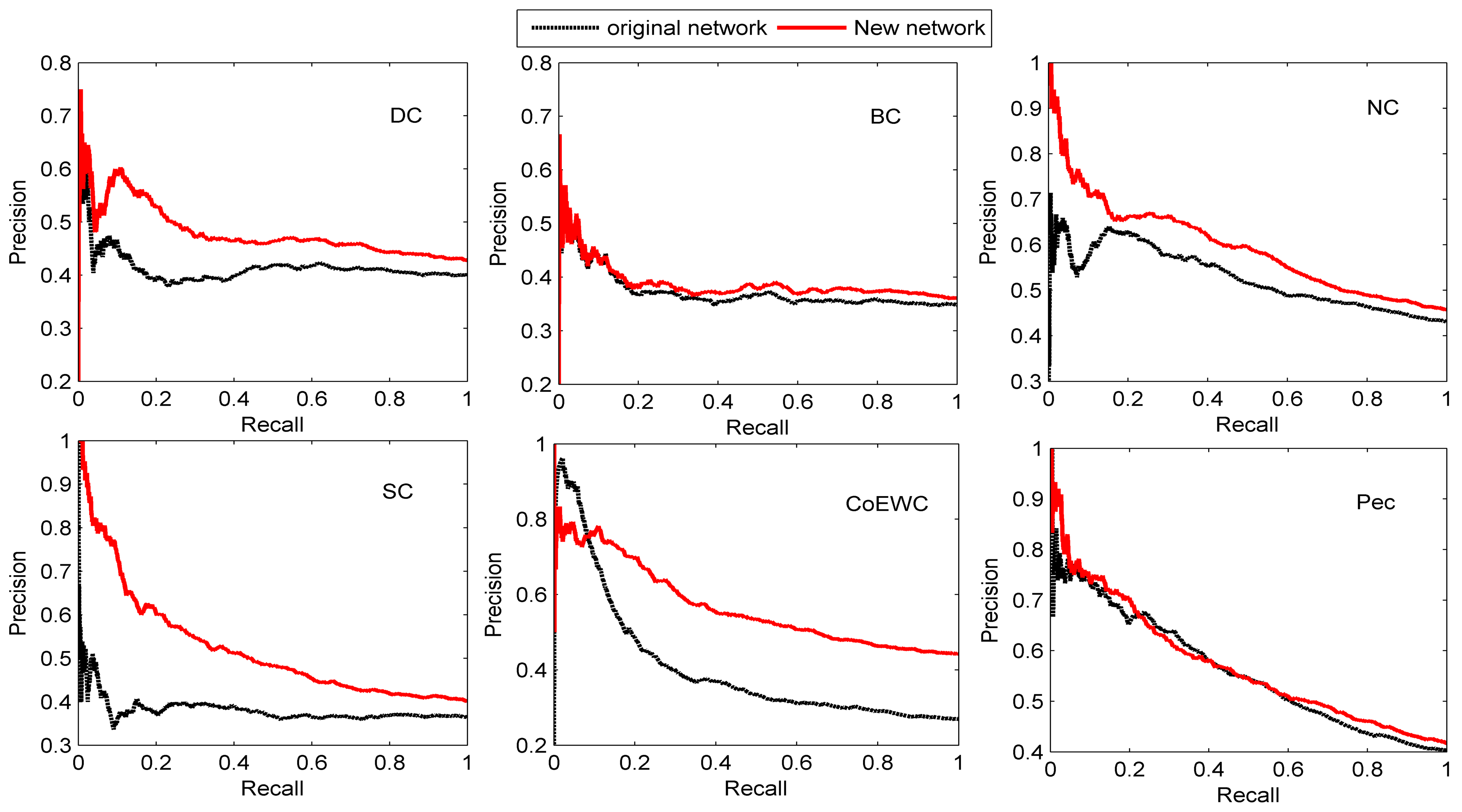

Supplement: S2 Fig — (TIF) [file pone.0177029.s016.tif]

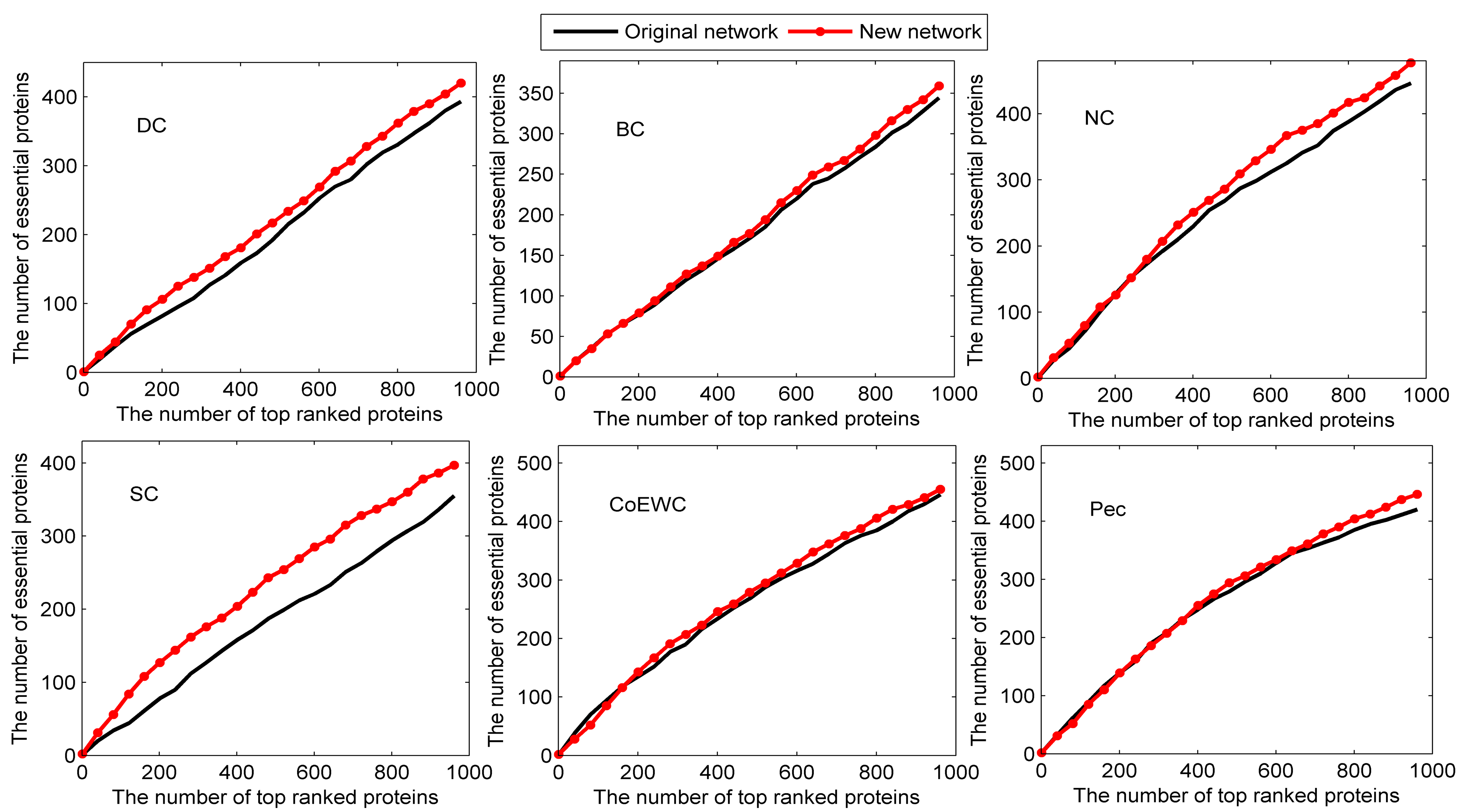

Supplement: S3 Fig — (TIF) [file pone.0177029.s017.tif]

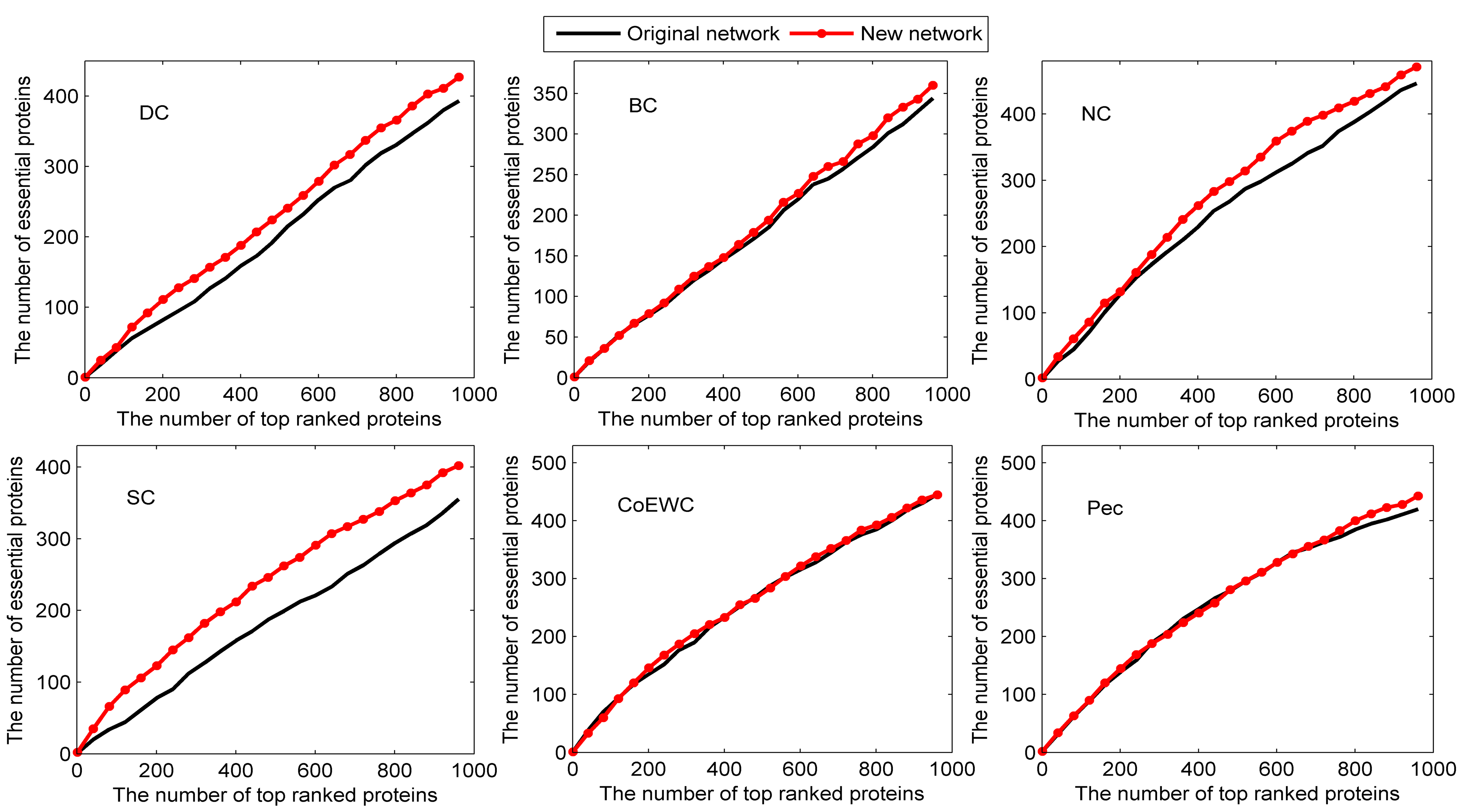

Supplement: S4 Fig — (TIF) [file pone.0177029.s018.tif]

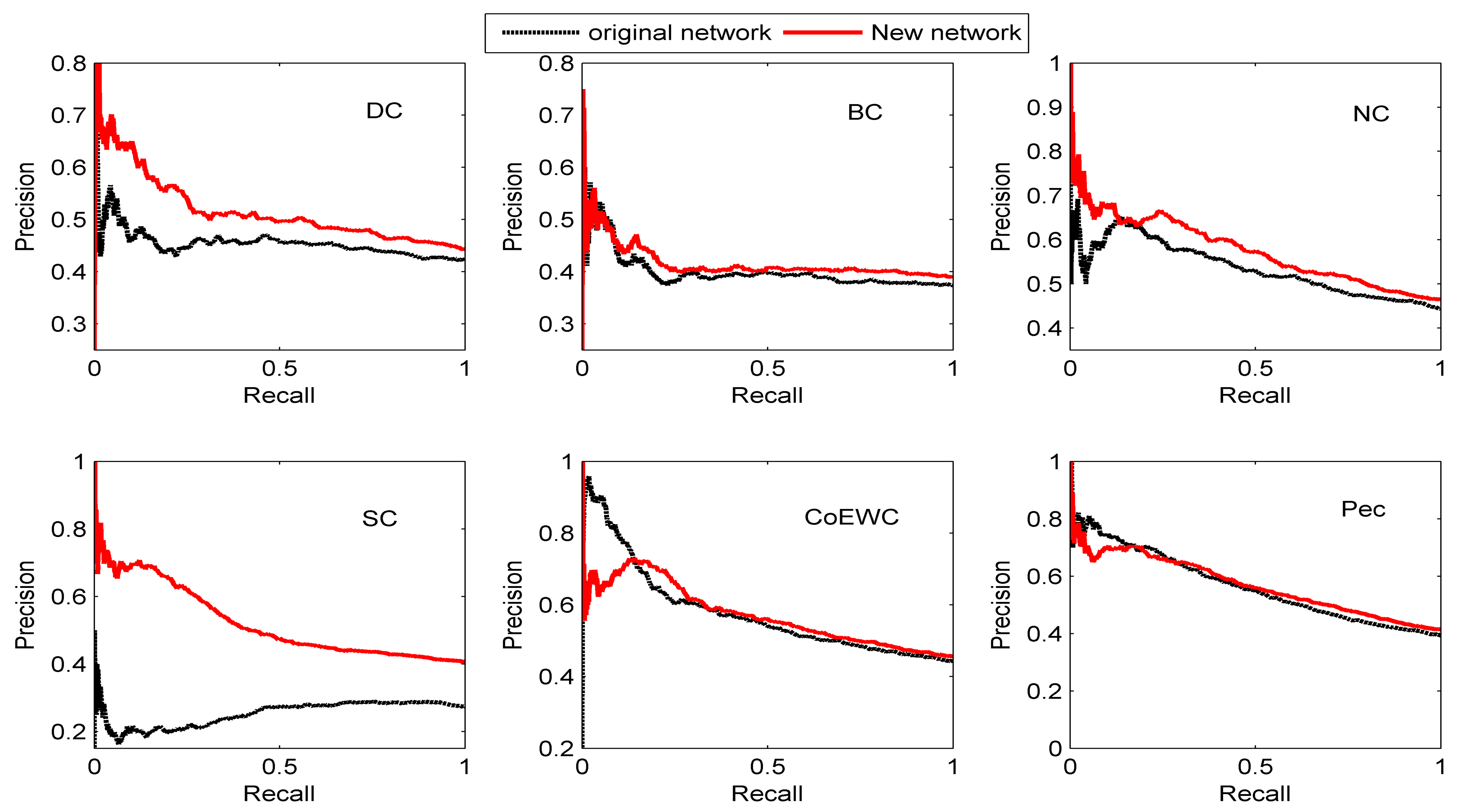

Supplement: S5 Fig — (TIF) [file pone.0177029.s019.tif]

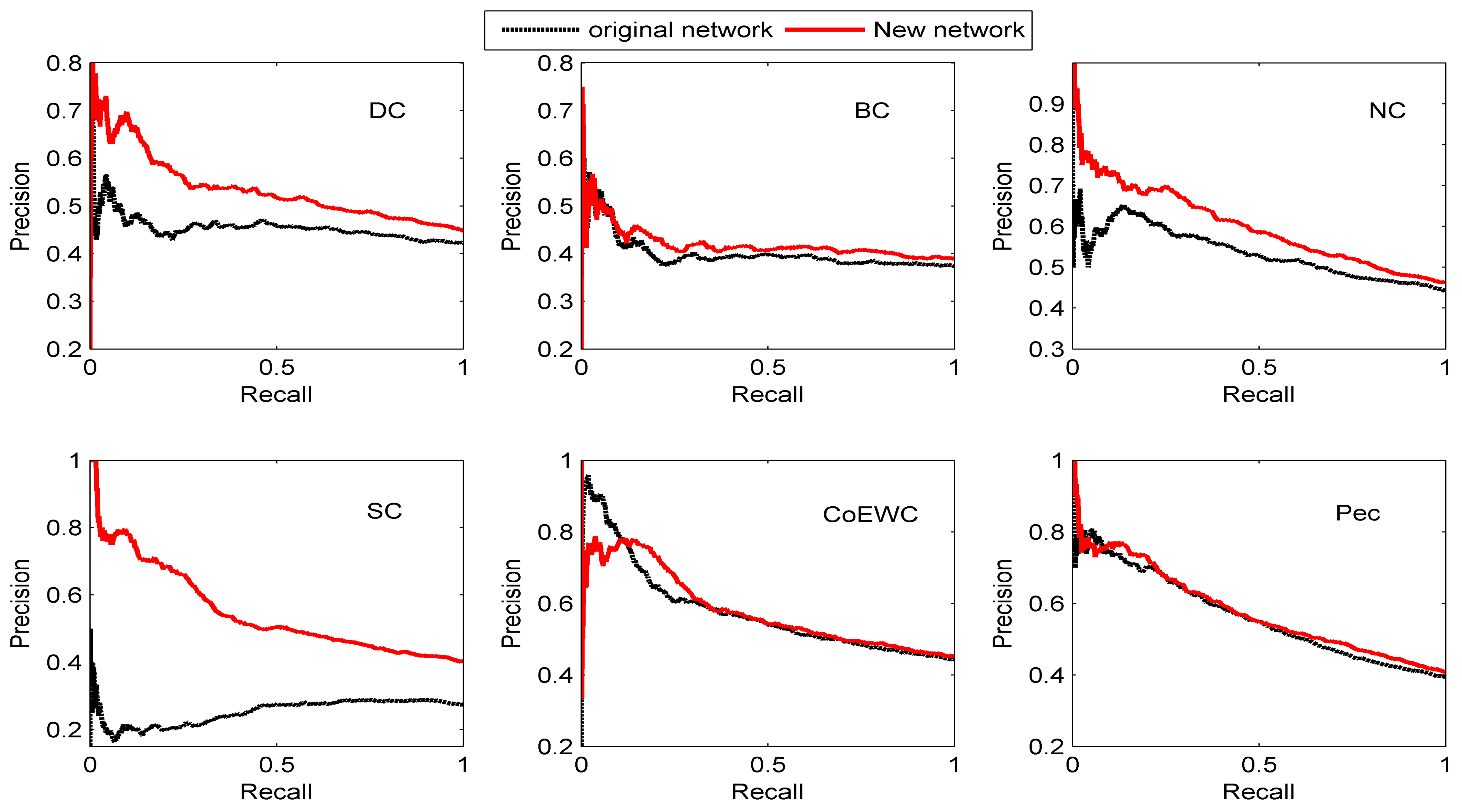

Supplement: S6 Fig — (TIF) [file pone.0177029.s020.tif]

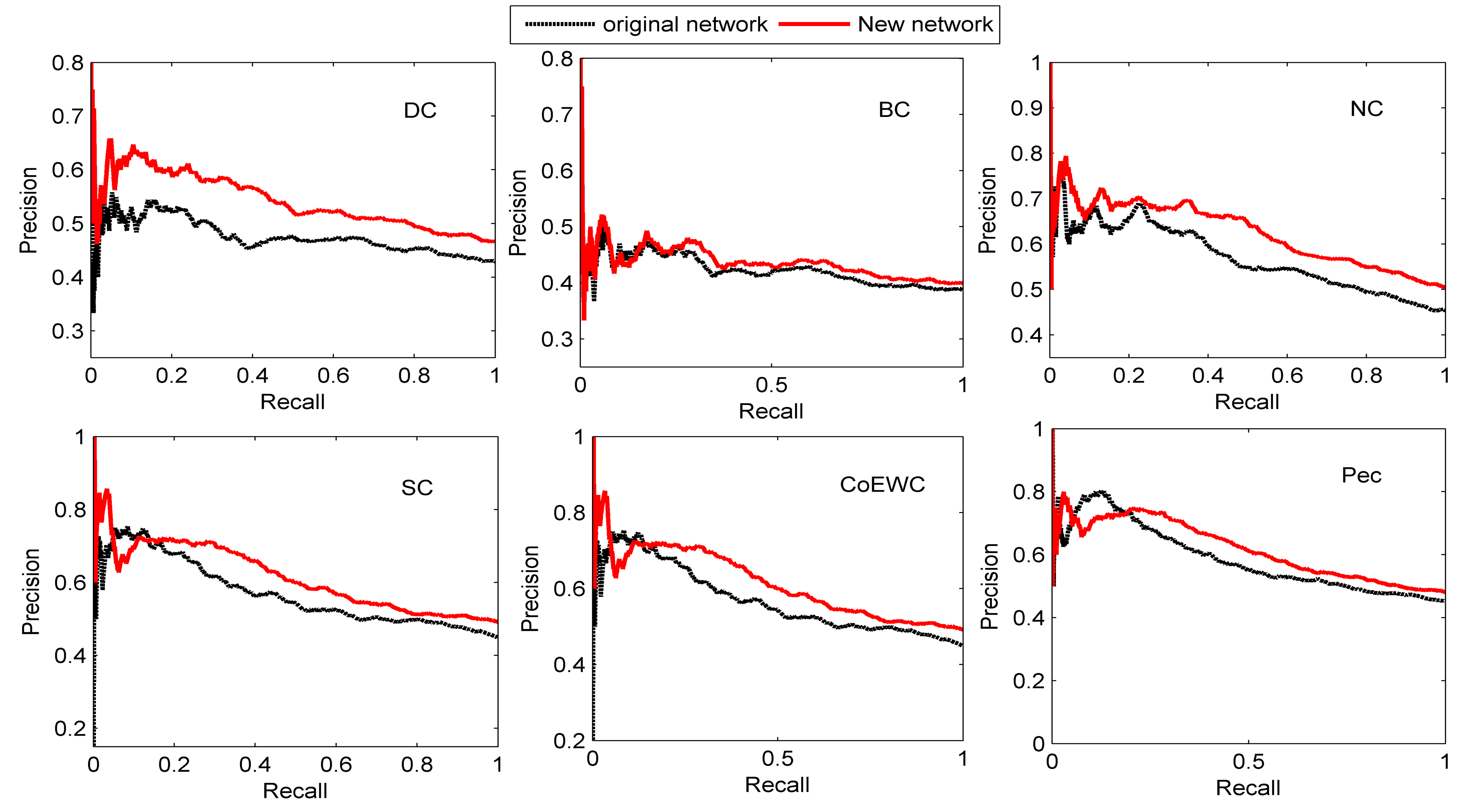

Supplement: S7 Fig — (TIF) [file pone.0177029.s021.tif]

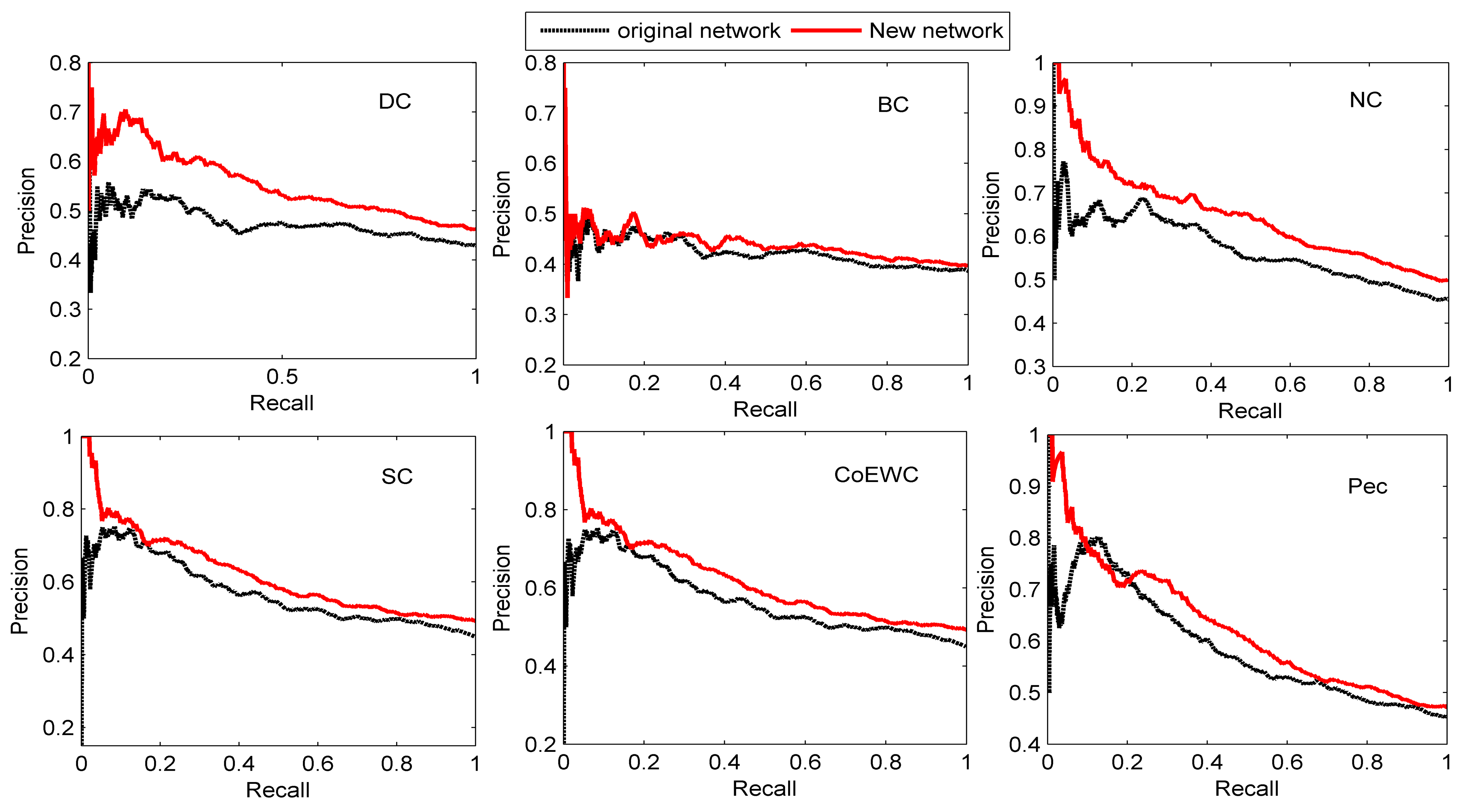

Supplement: S8 Fig — (TIF) [file pone.0177029.s022.tif]
